# MRCER-DR: Multi-Representation Confidence Entropy Rejection for IoT Open-Set Intrusion Detection

**DOI:** 10.3390/s26206436

**Published:** 2026-10-12

**Authors:** Ni Jin, Jiachun Zheng, Zhiping Xu, Chaopeng Li, Jiawen Yang

**Affiliations:** School of Ocean Information Engineering, Jimei University, Xiamen 361000, China; 202411810003@jmu.edu.cn (N.J.); zhipingxu@jmu.edu.cn (Z.X.); licp@jmu.edu.cn (C.L.); yangjiawen123@jmu.edu.cn (J.Y.)

**Keywords:** Internet of Things, open-set intrusion detection, multi-representation ensemble, joint rejection score

## Abstract

The rapid growth of Internet of Things (IoT) devices has made cyberattacks increasingly covert and evolving. Traditional closed-set intrusion detection systems fail to identify unknown attacks that are unseen during training. Existing open-set methods often rely on a single rejection signal, while multi-representation approaches mainly focus on feature fusion or decision aggregation rather than exploiting complementary uncertainty responses across representations. In addition, probability-based rejection may become less reliable under severe class imbalance. To address these issues, we propose Multi-Representation Confidence Entropy Rejection with Dual-Reject (MRCER-DR), an open-set intrusion detection method for IoT with low inference overhead. It constructs four representations (Raw, Graph, 2D-Stat, and Fusion) and builds a joint rejection score, Confidence Entropy Rejection (CER), combining the maximum class probability and prediction entropy. The Multi-Representation Confidence Ensemble (MRCE) then aggregates the CER scores across representations, while Dual-Reject supplements MRCE with the Mahalanobis distance under severe class imbalance. On UNSW-NB15, MRCER-DR achieves 83.1% unknown recall and an 86.2% AUROC; on ToN-IoT, these reach 97.7% and 90.9%. The single-sample inference latency ranges from 0.04 to 1.01 ms. Without modifying the classifier training, our method balances known-class classification and unknown attack detection, providing a low-overhead approach for IoT open-set intrusion detection.

## 1. Introduction

With the rapid development of Internet of Things (IoT) technologies in areas such as industrial control and smart manufacturing, the scale of IoT applications continues to expand across increasingly diverse intelligent environments. Consequently, the demand for security protection in these environments continues to increase. The increasing number of IoT devices and the growing degree of interconnection among devices have introduced significant security threats to IoT systems [1,2]. Therefore, developing efficient and reliable intrusion detection mechanisms has become an important research direction for ensuring the secure development of IoT [3]. Compared with traditional Internet environments, IoT networks typically exhibit strong device heterogeneity, complex protocol types, and resource-constrained nodes [4]. These characteristics necessitate security protection methods to consider the computational overhead and practical deployment conditions while maintaining detection performance [5]. An intrusion detection system (IDS) can identify potential attacks by analyzing network traffic or system behavior and has become an important component of IoT security protection systems [6].

Under the closed-set classification assumption, all traffic categories encountered during testing are assumed to be included in the training data [7]. Under this assumption, models mainly learn to distinguish known normal traffic from known attack categories and assign each test sample to one of the known classes. However, the attack patterns in real network environments continue to evolve, and new attacks, attack variants, and infrequent attacks may be completely absent during the training stage [8]. When encountering these unknown attacks, traditional closed-set classifiers still assign them to known classes, resulting in missed detections and reducing the ability of IDSs to provide early warnings for emerging attacks [9]. Therefore, compared with closed-set classification, open-set intrusion detection is more suitable for dynamic network environments. It requires models not only to accurately identify known traffic categories but also to effectively reject attack types that are unseen during training, thereby improving the unknown attack detection capabilities while maintaining the classification performance on known classes [10].

In recent years, research on open-set intrusion detection for unknown and zero-day attacks has gradually increased. Existing studies have expanded from unknown attack rejection to unknown class discovery and incremental learning [11], classification uncertainty estimation, and open-set traffic classification considering both detection performance and time complexity [10]. Previous studies have indicated that network traffic environments are highly dynamic, and improving model adaptability to emerging attacks and traffic variations has become an important direction in unknown attack detection research [12]. Meanwhile, some studies have introduced open-set recognition concepts into network intrusion detection tasks. These methods enable models to reject attacks that are absent during training while still identifying known attack categories, thereby providing a foundation for the subsequent analysis of new classes and model updates [13]. With the further development of this research area, the focus of open-set intrusion detection has gradually shifted from simply determining whether samples are anomalous to addressing more complex problems in open environments. For example, existing studies have begun to consider the differences among different unknown attack categories and investigate the impacts of changes in normal traffic caused by business and network environments on the detection results [14]. Other studies have combined known attack classification with abnormal traffic discovery, allowing different types of detection information to complement each other during zero-day attack identification [15].

These studies indicate that open-set intrusion detection requires not only determining whether test samples belong to unseen categories but also improving the reliability of unknown sample identification and effectively utilizing information from different sources to enhance the detection capabilities in complex network environments. These studies have promoted the development of IDSs from known attack classification toward unknown attack detection in open environments. However, for IoT scenarios, the stability of unknown sample identification and the effective utilization of diverse traffic information still require further investigation. First, some methods mainly rely on a single criterion for unknown sample identification. When this signal is affected by model overconfidence or distribution shifts, the rejection results may become unstable [16,17]. Second, although multi-representation information has been explored in intrusion detection, existing approaches mainly integrate different representations through feature fusion, reconstruction result aggregation, or voting decisions [18,19,20]. The complementary uncertainty responses produced by different representations have received relatively limited attention. Consequently, uncertainty information that may be weak in one representation but more evident in another is not fully utilized for unknown attack rejection. Finally, intrusion detection datasets usually exhibit significant class imbalance. Insufficient samples from minority classes may lead to unstable classification and further increase the difficulty of detecting certain challenging unknown attacks [21].

To address these challenges, this paper proposes an open-set intrusion detection method Multi-Representation Confidence Entropy Rejection with Dual-Reject (MRCER-DR) for IoT environments. The proposed method aims to improve the rejection capabilities for unknown attack categories while maintaining the classification performance on known attack categories. The proposed method consists of three main components: joint rejection scoring, multi-representation rejection information fusion, and conditional complementary rejection. Considering the computational and storage constraints of IoT edge environments, MRCER-DR adopts a lightweight design based on conventional classifier outputs, lightweight representation transformations, and postprocessing rejection scores. This design aims to improve unknown attack rejection while limiting the model complexity and inference overhead. Specifically, MRCER-DR adopts a machine learning classifier as the basis and represents network traffic through four representations: raw features, feature relationship graph features, lightweight two-dimensional statistical features, and fusion features. During the rejection stage, a progressive unknown attack rejection mechanism consisting of Confidence Entropy Rejection (CER), a Multi-Representation Confidence Ensemble (MRCE), and Dual-Reject is designed. CER combines the maximum class probability and prediction entropy to construct unknown sample rejection scores from the perspectives of prediction certainty and overall distribution uncertainty. Based on this, MRCE calculates CER scores under different representations and integrates rejection information from multiple representation spaces through normalization to improve the stability of unknown attack identification. Furthermore, Dual-Reject determines whether to introduce the Mahalanobis distance signal according to the imbalance degrees of known classes in the training set. By combining probability outputs with feature-space distances, it provides additional detection evidence for challenging unknown attacks under highly imbalanced scenarios.

MRCER-DR progressively integrates complementary rejection information through joint confidence entropy scoring, multi-representation uncertainty aggregation, and conditional feature-space rejection. The main contributions of this paper are summarized as follows:

(1) To address the limitation whereby a single confidence indicator cannot fully utilize the information in the prediction probability distribution, MRCER-DR combines the maximum class probability and prediction entropy within the CER score. Unlike methods that identify unknown samples only according to the maximum class probability, CER considers both the confidence of the predicted class and the uncertainty reflected by the complete prediction distribution, providing a more comprehensive basis for rejection. This method performs postprocessing based on classifier outputs without modifying the original training process of the classification model and introduces only limited additional computational overhead.

(2) To address the issue whereby rejection results based on a single representation are sensitive to the representation choice and cannot fully exploit diverse traffic information, MRCER-DR further integrates rejection information from the Raw, Graph, 2D-Stat, and Fusion representations at the score level. Instead of directly concatenating features from different representations, MRCER-DR integrates uncertainty information generated by raw features, feature relationship graph features, lightweight two-dimensional statistical features, and fusion features at the rejection score level, utilizing the diverse descriptions of unknown attacks provided by different representations. By aggregating the normalized CER scores, MRCE reduces the dependence of the rejection results on a single representation and improves the stability of unknown attack identification. In addition, the complementarity of multi-representation rejection information is further verified through a correlation analysis of the CER scores from different representations on known and unknown samples.

(3) To address the limitation of unknown attack detection under imbalanced scenarios, MRCER-DR supplements MRCE with the Mahalanobis distance through Dual-Reject. The Mahalanobis signal characterizes feature-space deviation and provides complementary information to the probability-based uncertainty captured by MRCE. Dual-Reject is conditionally activated according to the imbalance ratio of known classes in the training set. When the degree of class imbalance exceeds a predefined threshold, the Mahalanobis distance is introduced to assist MRCE in unknown sample identification. Otherwise, only MRCE is used for rejection. This mechanism does not utilize unknown-class information during testing. It selects the rejection strategy according to the class distribution of the training data, thereby improving the detection capabilities for challenging unknown attacks.

## 2. Related Work

Early intrusion detection mainly relied on rule matching and expert knowledge. Based on the detection mechanisms, existing intrusion detection methods can generally be divided into misuse detection and anomaly detection. Misuse detection identifies known threats by matching predefined attack patterns and usually achieves high detection accuracy, but it is not able to handle continuously evolving attack behaviors. Anomaly detection identifies anomalies by analyzing deviations between network traffic and normal behavior patterns, showing greater potential for unknown attack detection [22]. With the increasing complexity of network traffic and attack types, machine learning algorithms such as decision trees [23], random forest [24], support vector machines [25], and XGBoost [26] have gradually been applied to intrusion detection systems. These methods mainly perform classification based on filtered or constructed traffic statistical features and usually have lower training and inference costs, making them suitable for structured traffic analysis and resource-constrained environments. In contrast, CNNs [27] can extract local patterns from traffic data, while RNNs and LSTM networks [28] are suitable for analyzing traffic behaviors with temporal dependencies. Autoencoders [29] can learn compact representations of network traffic for subsequent intrusion classification. Transformers [30] and GNNs [31] are, respectively, used to capture long-range dependencies and relationships among communication entities. These deep learning methods enhance the ability of models to learn complex attack patterns, but they usually introduce higher computational and storage costs. Therefore, their deployment on IoT edge devices still requires a balance between detection performance and operational efficiency. More importantly, most existing methods are still developed under the closed-set assumption, where the training and testing categories are consistent. Although they can effectively identify known attacks, they struggle to handle new attacks that are absent during training [32].

Supervised intrusion detection systems usually learn classification boundaries based on predefined traffic categories during training and assume that the training and testing sets share the same class space. When the test samples originate from attack categories that are unseen during training, traditional classifiers still assign them to existing classes. Therefore, open-set intrusion detection requires not only distinguishing known traffic categories but also determining whether input samples belong to known classes and rejecting potential unknown attacks. Shieh et al. [33] introduced reciprocal point learning into DDoS attack detection and used open-set classification to simultaneously handle known attacks and unknown DDoS attacks that were absent during training. Cevallos et al. [34] proposed HERO, which further extends unknown attack detection to high-dimensional raw network traffic. The method adopts a two-stage training strategy that separates traffic representation learning from the discrimination process to detect previously unseen zero-day attacks. Methods based on classifier outputs provide another approach for unknown attack detection. They usually construct rejection scores from the prediction results, including the maximum class probability, prediction entropy, and calibrated prediction uncertainty. Maximum softmax probability (MSP) is a classical output confidence scoring method that measures the prediction confidence based on the maximum class probability from classifier outputs and accordingly distinguishes in-distribution samples from out-of-distribution samples [35]. Matejek et al. [36] used MSP as an out-of-distribution (OOD) detection baseline in group-level network intrusion detection experiments and considered low maximum class probabilities as potential indicators of zero-day attacks. Talpini et al. [16] used the Shannon entropy of prediction distributions to measure the overall prediction uncertainty and further proposed an uncertainty-calibrated Bayesian neural network, in which traffic samples distant from the training distribution exhibit higher uncertainty. Li et al. [37] proposed DEMTR based on evidential deep learning, which simultaneously estimates class probabilities and prediction uncertainty, calibrates uncertainty through a dedicated loss function, and rejects unknown malicious traffic accordingly. The maximum class probability mainly reflects the prediction confidence of the most likely class, while prediction entropy describes the concentration or dispersion of the complete class probability distribution. Both provide different perspectives for characterizing prediction uncertainty.

In the field of intrusion detection, some studies have also applied methods such as OpenMax and energy scores for zero-day attack identification. Fang et al. [38] introduced OpenMax into an InfoGAN-based open-set detection framework. The method identifies unknown attacks by recalibrating the activation scores of known classes and estimating unknown-class probabilities. Another category of methods constructs energy scores from classifier outputs. Liu et al. [39] proposed an energy-based OOD detection method that distinguishes in-distribution and OOD samples using energy values, alleviating the overconfidence problem of Softmax scores for certain OOD samples. Baye et al. [40] proposed the varMax framework, which integrates Softmax score differences, logit variance, and energy scores to further analyze classification results and enhance the ability to identify zero-day attacks and unknown network activities. These studies demonstrate that postprocessing scores constructed from classifier outputs can provide effective evidence for unknown attack rejection without significantly modifying the original classification model.

Beyond prediction outputs, some methods identify unknown attacks by analyzing the deviation between samples in the feature-space and known-class distributions. Lee et al. [41] estimated class-specific feature means and shared covariance matrices based on class-conditional Gaussian distributions. They then used the Mahalanobis distance to construct confidence scores and determine whether test samples deviated from known-class distributions. Yu et al. [32] combined a bidirectional gated recurrent unit (BiGRU) with prototype learning to obtain more discriminative feature representations by improving the intra-class compactness and inter-class separation of known categories. They further employed a Weibull model based on extreme value theory to identify unknown industrial network intrusions. Zhao et al. [42] assigned a dedicated autoencoder to each known traffic category, used category-specific feature manifolds generated by autoencoders to replace traditional class prototypes, and identified unknown malicious encrypted traffic according to the matching scores between samples and class representations. Meng et al. [43] proposed OC-MAL, which combines Deep SVDD with an autoencoder. The method uses distance constraints to gather known traffic around the hypersphere center, preserves class information through reconstruction errors, and identifies unknown malicious traffic based on joint anomaly scores. Overall, methods based on prediction outputs can evaluate uncertainty with a low computational cost, while feature-space distance methods provide intuitive measures of geometric deviation. However, when applied independently, both types of methods can be affected by factors such as model overconfidence and limited information from individual signals, resulting in unstable rejection performance in complex network traffic environments.

In recent years, some studies have explored the use of uncertainty information from multiple representations to assist decision-making and reduce the uncertainty caused by single rejection signals. Liu et al. [44] proposed a trustworthy multi-view deep learning method that integrates predictive evidence from different views to reduce uncertainty and improve prediction reliability. Jung et al. [45] further addressed uncertainty estimation in multi-view data by modeling uncertainty from different views separately and integrating the prediction results to improve out-of-distribution sample detection. Xie et al. [46] investigated uncertainty utilization under incomplete multi-view conditions and enhanced multi-view fusion by estimating the reliability of different views. These studies indicate that prediction information from different representation spaces is complementary, and integrating multi-representation uncertainty can improve decision reliability.

In intrusion detection, existing studies have attempted to construct multiple representations from different perspectives of network traffic to better characterize complex attack behaviors. Network traffic can be represented through various means, such as statistical features, structural relationships, and temporal patterns, with each representation emphasizing different types of information. He et al. [18] divided features within the same traffic record into multiple hierarchical views, used a multimodal deep autoencoder to learn joint representations, and employed LSTM to extract temporal relationships among adjacent network connections for feature-level fusion. Nie et al. [19] proposed M2VT IDS, which constructs three views from IoT traffic, including spatiotemporal sequences, header field patterns, and payload semantics. The information from these views is integrated through a multi-view shared network and a task-specific attention mechanism to support anomaly detection and attack identification. Nandi et al. [20] proposed MVNIDS, which constructs network, flow, and image views from raw packets; trains view-specific autoencoders separately; and combines reconstruction decisions from different views through majority voting to improve the detection capabilities for zero-day attacks and attack variants. Existing multi-representation methods mainly improve the detection performance through joint feature learning, reconstruction result aggregation, or voting decisions. However, studies that fuse rejection scores and uncertainty responses across different representations remain relatively limited.

In summary, intrusion detection research has gradually expanded its focus from improving known attack classification capabilities to unknown attack identification, leading to technical directions including prediction uncertainty estimation, feature-space anomaly measurement, and multi-representation fusion. As summarized in Table 1, these approaches provide different sources of information for unknown attack detection, but their emphasis differs across classifier output uncertainty, feature-space deviation, and multi-representation integration. In particular, the use of complementary uncertainty responses across different traffic representations for rejection remains relatively limited. To address this gap, MRCER-DR combines the maximum class probability and prediction entropy to construct rejection scores, integrates uncertainty information across multiple traffic representations, and conditionally introduces the feature-space distance as supplementary rejection information under severe class imbalance.

## 3. The Proposed Model

Our MRCER-DR aims to improve the rejection capabilities for attack classes that are unseen during training while maintaining the classification performance on known classes. Given a training set containing only samples from known classes, the model is required to correctly classify known samples during testing and identify unknown attack samples as unknown. Unlike traditional closed-set intrusion detection, test samples in an open-set scenario do not necessarily belong to attack classes included during training. Therefore, the model cannot rely solely on closed-set classification results and also requires rejection signals that reflect the reliability of class assignment.

The core idea of MRCER-DR is to extend unknown attack rejection from a decision based on a single maximum class probability to a joint decision process using multiple representations and multiple signals. A single confidence score cannot fully reflect the uncertainty contained in the classifier output distribution, while a single representation may fail to capture the abnormal behavior of unknown attacks across different feature spaces. To address this issue, a joint confidence entropy rejection score is first designed by combining the maximum class probability with the prediction entropy to characterize sample uncertainty under a single representation in the open-set scenario. Based on this design, the model further constructs four complementary representations, namely Raw, Graph, 2D-Stat, and Fusion. CER scores are calculated separately in the corresponding representation spaces and then integrated after normalization to obtain the MRCE score, thereby improving the stability of unknown sample rejection. In intrusion detection datasets with highly imbalanced class distributions, the classifier may produce overconfident predictions for some unknown attack samples. As a result, probability-based rejection may be insufficient for identifying these challenging unknown classes. To address this issue, MRCER-DR further introduces the Dual-Reject mechanism, which combines the probability-space uncertainty signal provided by MRCE with the feature-space deviation signal provided by the Mahalanobis distance. When the known-class distribution in the training set is highly imbalanced, Dual-Reject is activated to supplement the probability-based rejection signal; otherwise, only MRCE is used for open-set discrimination. This activation strategy relies only on class statistics from the training set and does not use any information from the test set.

**Figure 1:**
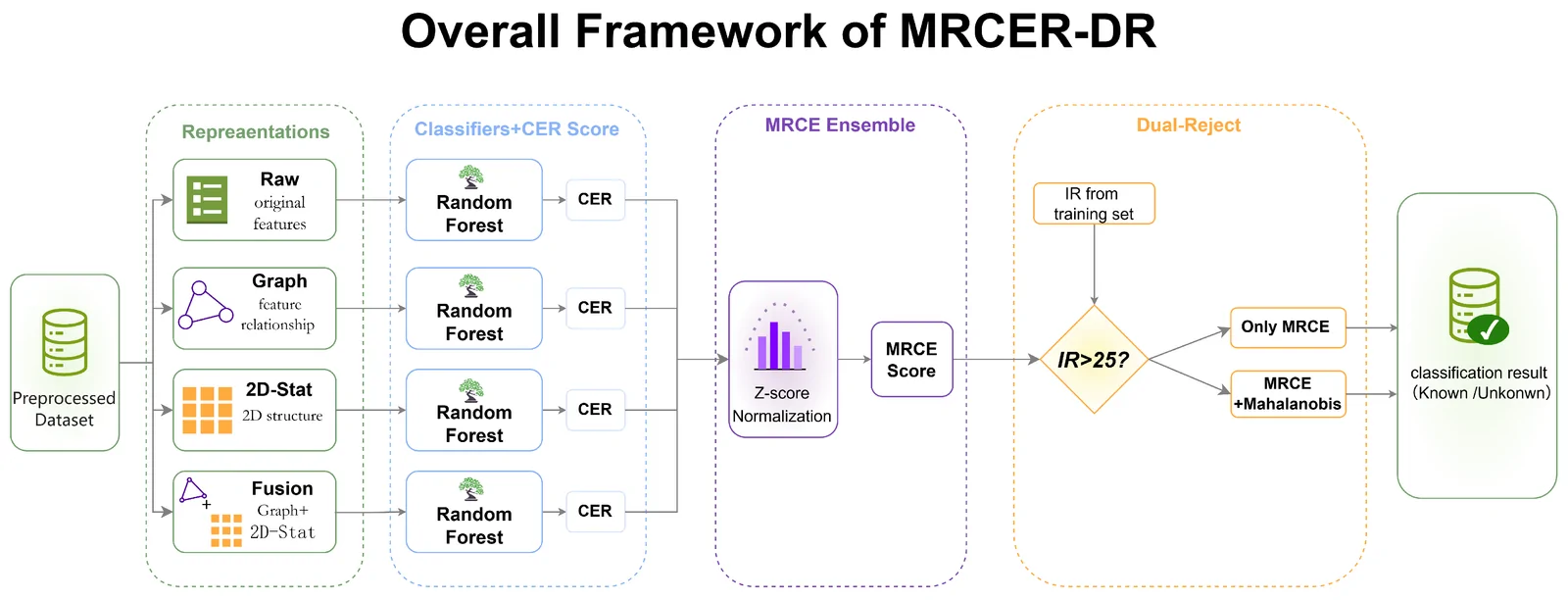
Overall framework of MRCER-DR.

As shown in Figure 1, MRCER-DR consists of three progressive modules. CER constructs the basic rejection score, MRCE integrates uncertainty information from multiple representations, and Dual-Reject further introduces a feature-space distance constraint under severe class imbalance. Since these modules are mainly based on postprocessing scores, the proposed model does not require complex additional model structures and is therefore suitable for IoT intrusion detection scenarios that are sensitive to the inference overhead.

### 3.1. Joint Confidence Entropy Rejection Score (CER)

In open-set intrusion detection, the model not only correctly classifies known samples but also determines whether a test sample may originate from an attack class that was absent during training. Traditional rejection methods based on the maximum class probability usually use the highest output probability of the classifier to determine whether a sample belongs to a known class. However, the maximum class probability only reflects the strength of the model response to the most likely class and cannot describe how the remaining probability is distributed across other classes. When an unknown attack sample is similar to a known class, the classifier may still assign it a high maximum class confidence, causing the unknown sample to be misclassified as a known class.

To more comprehensively characterize uncertainty in classifier outputs, the Confidence Entropy Rejection (CER) score combines the maximum class probability and prediction entropy into a joint rejection score. The maximum class probability reflects the confidence of the model in the most likely class, while prediction entropy describes the dispersion of the probability distribution across all known classes. These two terms describe sample uncertainty from the perspectives of the maximum class response and the overall distribution uncertainty, respectively.

Given the predicted probability distribution $p\left(x\right)=[p_{1}\left(x\right),p_{2}\left(x\right),\ldots ,p_{K}\left(x\right)]$ of sample *x* over *K* known classes, CER first computes the complement of the maximum class probability and the normalized prediction entropy, as shown in Equations (1) and (2):

(1) $$u_{\mathrm{conf}}\left(x\right)=1-\max_{j=1,\ldots ,K}p_{j}\left(x\right)$$

(2) $$H=-\frac{1}{\log(\max(K,2))}\sum_{j=1}^{K}p_{j}\left(x\right)\log\left(p_{j}\left(x\right)+\epsilon \right)$$

where ϵ is a numerical stability term. The weighted combination of these two terms is defined as the CER score, as shown in Equation (3):

(3) $$s_{\mathrm{CER}}\left(x\right)=\alpha \cdot u_{\mathrm{conf}}\left(x\right)+\left(1-\alpha \right)H$$

where α∈[0,1] controls the relative contributions of the inverse maximum class probability term and the normalized prediction entropy. A higher CER score indicates greater uncertainty and therefore a higher likelihood that the sample belongs to an unknown class.

The CER threshold is determined using a validation set containing only known classes. The model uses stratified sampling by class to select 20% of the known training samples as the validation set and calculates the CER scores for these validation samples. The 0.8 quantile of the CER scores on the validation set is used as the rejection threshold $\tau_{\mathrm{CER}}$. As a postprocessing rejection score, CER does not modify the structure of the underlying classifier and serves as the basis for the subsequent multi-representation rejection mechanism.

### 3.2. Multi-Representation Confidence Ensemble (MRCE)

Network traffic samples generally consist of various features, such as statistical features, protocol features, and connection behavior features. Different types of attacks are often characterized not only by abnormal values of individual features but also by combined variations across multiple features. If rejection decisions rely on only a single representation space, the uncertainty signal may be limited by the corresponding feature perspective, making some unknown attack samples difficult to distinguish effectively. To address this limitation, this paper proposes the Multi-Representation Confidence Ensemble (MRCE), which calculates the CER scores separately in multiple complementary representation spaces to improve the stability of open-set rejection.

Four representations are constructed as inputs to MRCE: Raw, Graph, 2D-Stat, and Fusion. The Raw representation directly uses the preprocessed original features to preserve the basic statistical information of network traffic samples. The Graph representation extends the original features from the perspective of feature relationships to incorporate association information among different traffic features. The 2D-Stat representation rearranges one-dimensional tabular features using a fixed layout and extracts statistical aggregation information along rows and columns, thereby providing an additional grouped statistical perspective. The Fusion representation further concatenates the Graph and 2D-Stat representations, allowing the classifier to utilize both feature relationship information and lightweight two-dimensional statistical information.

For the Graph representation, each feature dimension is treated as a node in the graph, and a feature relationship graph is constructed according to the feature correlation and supervised dependency strength.

Let $\rho_{ij}$ denote the Pearson correlation coefficient between features *i* and *j*, and let $a_{i}$ denote the normalized supervised dependency strength between feature *i* and the class label. Both $\rho_{ij}$ and $a_{i}$ are calculated only from the known-class training data. The resulting adjacency matrix is kept fixed during testing, and no test label information is used in this process. The edge weight is defined as shown in Equation (4):

(4) $$W_{ij}=\frac{1}{2}\left|\rho_{ij}\right|+\frac{1}{2}\sqrt{a_{i}a_{j}}.$$

Subsequently, only the neighbors with the highest weights are retained for each node, and the adjacency matrix is symmetrized. Following the commonly used graph convolutional network formulation that adds self-loops and applies symmetric normalization [47], self-loops are further added and the adjacency matrix is normalized. Let the processed adjacency matrix be *A* and the degree matrix be *D*. The normalized adjacency matrix is defined as shown in Equation (5):

(5) $$\hat{A}=D^{-\frac{1}{2}}AD^{-\frac{1}{2}}$$

Two propagation layers are then applied to the feature correlation graph, and the resulting features are concatenated with the original features to obtain the Graph representation. This transformation incorporates feature association information while maintaining a relatively simple representation construction process.

For the 2D-Stat representation, given a feature vector *x* of length *d*, let $r=\left\lfloor \sqrt{d}\right\rfloor$ and $c=\left\lceil \frac{d}{r}\right\rceil$. When rc>d, zeros are padded at the end of the feature vector until its length reaches rc, after which it is rearranged row by row into a two-dimensional matrix $M\in R^{r\times c}$ of size r×c. For this matrix, the row means $m_{\mathrm{row}}\left(x\right)$, column means $m_{\mathrm{col}}\left(x\right)$, row maxima $q_{\mathrm{row}}\left(x\right)$, and column maxima $q_{\mathrm{col}}\left(x\right)$ are calculated separately. The final 2D-Stat representation is obtained by concatenating the original features with these statistics, as shown in Equation (6):

(6) $$\phi_{2D\text{-}\mathrm{Stat}}\left(x\right)=\left[x,\;m_{\mathrm{row}}\left(x\right),\;m_{\mathrm{col}}\left(x\right),\;q_{\mathrm{row}}\left(x\right),\;q_{\mathrm{col}}\left(x\right)\right].$$

The row and column means and maxima provide grouped statistical summaries that complement the original per-feature information, while the original feature vector is retained in the representation. The feature order produced by the preprocessing is kept fixed when constructing the 2D-Stat representation. The representation used in this study is intended to provide additional grouped statistical information for tabular traffic data.

The Fusion representation integrates the information contained in the Graph and 2D-Stat enhanced representations, as defined in Equation (7):

(7) $$\phi_{\mathrm{fusion}}\left(x\right)=\left[\phi_{2D\text{-}\mathrm{Stat}}\left(x\right),\;\phi_{\mathrm{graph}}\left(x\right)\right]$$

A random forest (RF) classifier is trained separately for each representation r∈{Raw, Graph, Fusion, 2D-Stat}. For a given test sample *x*, the RF classifier associated with each representation outputs a class probability distribution, from which the corresponding CER score is calculated. Since CER scores from different representations may have different numerical ranges and distributions, the CER score of each representation is standardized using Z-score normalization, as shown in Equation (8):

(8) $${\hat{s}}_{r}\left(x\right)=\frac{s_{r}\left(x\right)-\mu_{r}}{\sigma_{r}+\epsilon }$$

where $s_{r}\left(x\right)$ is the CER score of sample *x* under representation *r*; $\mu_{r}$ and $\sigma_{r}$ denote the mean and standard deviation of the CER scores under this representation, respectively; ϵ is a numerical stability term; and ${\hat{s}}_{r}\left(x\right)$ is the standardized CER score. The four standardized CER scores are then averaged to obtain the MRCE score of the sample.

The MRCE threshold is also determined using the known-class validation set.

The MRCE scores of the validation samples are calculated, and their 0.8 quantile is used as the rejection threshold $\tau_{\mathrm{MRCE}}$. The 0.8 quantile is adopted as an intermediate setting to balance unknown-sample rejection and known-class classification performance, as further examined in the threshold sensitivity analysis in Section 4.10. During testing, the predicted label for known classes is produced by the classifier trained on the Fusion representation. Let $s_{\mathrm{MRCE}}\left(x\right)$ denote the MRCE ensemble score. If $s_{\mathrm{MRCE}}\left(x\right)>\tau_{\mathrm{MRCE}}$, the sample is classified as unknown; otherwise, the known-class label produced by the Fusion classifier is retained.

Through this design, MRCE separates known-class classification from unknown-class rejection. The Fusion representation is mainly used to provide the classification result for known classes, while MRCE aggregates uncertainty signals from multiple representation spaces for open-set rejection. Compared with CER based on a single representation, MRCE exploits the complementarity among different feature perspectives, allowing abnormal uncertainty exhibited by unknown attack samples in particular representation spaces to contribute to the final rejection decision.

To further explain the role of multi-representation integration, a representation correlation analysis is introduced in the experimental evaluation. The effectiveness of MRCE does not simply arise from increasing the number of representations but depends on whether different representations provide complementary uncertainty signals for unknown samples. If the CER rankings of multiple representations are highly consistent for unknown samples, simple averaging provides limited additional discriminative information. In contrast, if different representations remain relatively consistent for known samples but show more distinct ranking differences for unknown samples, this indicates that they capture uncertainty in unknown attacks from different perspectives.

### 3.3. Dual-Reject

MRCE strengthens the rejection signal in the probability space by integrating CER scores from multiple representations, but it still relies on class probabilities produced by the classifiers. When an unknown sample strongly overlaps with a known class in the prediction probability space, the model may still produce high confidence, making it difficult for MRCE to reject the sample. Previous studies have shown that class imbalance may cause samples from tail known classes to be more easily classified as unknown, while out-of-distribution (OOD) samples are more likely to be biased toward head known classes, thereby reducing the separability between known and unknown samples [48]. Therefore, in intrusion detection datasets with highly imbalanced class distributions, relying solely on probability outputs may be insufficient for identifying certain unknown attacks. To compensate for the limitations of probability-space rejection, this paper introduces Dual-Reject, a dual-signal rejection mechanism. Dual-Reject combines the probability-space uncertainty signal provided by MRCE with the feature-space deviation signal provided by the Mahalanobis distance. MRCE measures the prediction uncertainty across multiple representations, whereas the Mahalanobis distance measures whether a sample deviates from the feature-space distributions of known classes. The two signals characterize the likelihood of a sample being unknown from the perspectives of probability outputs and feature distributions and therefore provide complementary information.

Specifically, the Mahalanobis score is calculated in the Fusion representation space. For each known class *c*, the model estimates the mean vector $\mu_{c}$ and regularized covariance matrix $\Sigma_{c}$ using the training samples of that class. The Mahalanobis distance between a test sample *x* and class *c* is calculated as shown in Equation (9):

(9) $$d_{c}\left(x\right)=\sqrt{{\left(x-\mu_{c}\right)}^{T}\Sigma_{c}^{-1}\left(x-\mu_{c}\right)}$$

The minimum distance from the test sample to all known-class distributions is used as the feature-space anomaly score. A larger distance indicates that the sample is farther from all known-class distributions and is therefore more likely to belong to an unknown class. To improve the numerical stability of covariance estimation, a regularization term is added to the covariance matrix, and the Mahalanobis scores are normalized.

The class-specific statistics required for Mahalanobis distance calculation are determined during training and remain fixed during subsequent inference. Specifically, the class mean vectors, regularized covariance matrices, and their inverse matrices are computed from the known-class training data and stored for subsequent inference. Let *C* denote the number of known classes and let $d_{f}$ denote the dimensionality of the Fusion representation. The inversion of the *C* class-specific $d_{f}\times d_{f}$ covariance matrices has computational complexity of $O(Cd_{f}^{3})$ and is performed once during training. For each test sample, the model calculates its Mahalanobis distance to all known classes with per-sample complexity of $O(Cd_{f}^{2})$.

Dual-Reject uses an OR rule to combine the MRCE and Mahalanobis rejection signals. Let the normalized Mahalanobis score be $s_{\mathrm{Maha}}\left(x\right)$. The final rejection rule is defined as shown in Equation (10):

(10) $$\mathrm{reject}\left(x\right)=\left[s_{\mathrm{MRCE}}\left(x\right)>\tau_{\mathrm{MRCE}}\right]\vee \left[s_{\mathrm{Maha}}\left(x\right)>\tau_{\mathrm{Maha}}\right]$$

Here, $\tau_{\mathrm{MRCE}}$ is determined by the 0.8 quantile of the MRCE scores on the validation set, while $\tau_{\mathrm{Maha}}$ is determined by the 0.95 quantile of the Mahalanobis scores on the validation set. The stricter Mahalanobis quantile is used because this branch serves only as a supplementary rejection signal under the OR rule, helping to limit additional false rejections of known samples. A sample is labeled as unknown whenever either MRCE or the Mahalanobis signal identifies it as abnormal. The purpose of this module is not to replace MRCE with the Mahalanobis distance but to introduce feature-space deviation as a complementary signal when MRCE cannot effectively reject certain high-confidence unknown samples.

Since the Mahalanobis distance may introduce unnecessary false rejections on datasets with relatively balanced class distributions, Dual-Reject is not activated unconditionally for all datasets. The model first calculates the class imbalance ratio using the numbers of samples from known classes in the training set. In this study, 25 is used as a fixed heuristic activation threshold, serving as a simple criterion for restricting the supplementary Mahalanobis branch to clearly imbalanced training distributions. When the ratio between the largest and smallest classes exceeds 25, Dual-Reject is activated; otherwise, only MRCE is used for rejection. This value is not intended to represent a universally applicable imbalance boundary, and its applicability to different class distributions requires further investigation. This activation rule relies only on class statistics from the training data and does not use any information from the test set. Through this conditional activation mechanism, MRCER-DR retains the simple MRCE rejection process under general conditions while introducing an additional feature-space signal under class imbalance to improve the detection of challenging unknown attack classes.

## 4. Experiments and Results

### 4.1. Datasets and Data Preprocessing

To evaluate the open-set intrusion detection capabilities of MRCER-DR under different network environments and attack distributions, two publicly available IDS datasets are selected for the experiments: UNSW-NB15 [49] and ToN-IoT [50]. These datasets cover both traditional enterprise networks and IoT networks, allowing the applicability of the proposed model to be evaluated from different perspectives.

The UNSW-NB15 dataset was developed by the Australian Centre for Cyber Security and is a widely used benchmark dataset in intrusion detection research. The dataset contains normal traffic and multiple attack types, such as Fuzzers, Analysis, Backdoors, and DoS, representing a relatively complex distribution of network attacks. Compared with some earlier datasets, UNSW-NB15 contains basic traffic features, content features, temporal features, and connection statistics and is therefore commonly used to evaluate intrusion detection models in multi-class attack classification tasks. Previous studies have noted certain differences in the class proportions and feature-space distributions between its official training and testing partitions [51,52]. In addition, some studies have adopted alternative data splitting strategies rather than directly using the official partition [53,54]. Based on these considerations, our study combines the official training and testing sets and then performs stratified sampling, with 70% used for training and 30% for testing. Additional results obtained using the official UNSW-NB15 partition are provided in Appendix A. Since the dataset exhibits a clearly imbalanced class distribution, with relatively few samples in some attack classes, it can be used to evaluate the open-set rejection capabilities of MRCER-DR under class imbalance.

The ToN-IoT dataset was developed for IoT and Industrial Internet of Things (IIoT) security scenarios and contains multi-source information, including network traffic, operating system logs, telemetry data, and IoT/IIoT device data. In this study, we use the network traffic data from ToN-IoT. The dataset covers categories such as normal, backdoor, and DDoS, representing common attack behaviors in IoT/IIoT networks. Compared with UNSW-NB15, ToN-IoT places a greater emphasis on cybersecurity threats involving IoT devices and industrial control environments, making it suitable for evaluating the open-set detection capabilities of MRCER-DR in IoT scenarios. The ToN-IoT dataset is randomly split using stratified sampling, with 70% used as the training set and 30% as the testing set.

A unified data preprocessing procedure is applied in all experiments. First, the original field names and class labels are standardized. All column names are converted to lowercase, and redundant spaces and special characters are removed. Labels representing normal traffic, such as normal and benign, are uniformly mapped to normal. Second, identifier fields that are weakly related to attack discrimination or may introduce data leakage are removed, including flow IDs, timestamps, and source/destination IP addresses. For numerical features, missing values, infinite values, and invalid numerical values are replaced with 0, and RobustScaler is applied for feature scaling to reduce the influence of extreme values on the feature distributions. Low-cardinality categorical features are converted into numerical vectors using one-hot encoding. Finally, all class labels are encoded as integer IDs for subsequent classifier training and open-set evaluation.

Under the open-set setting, two attack classes from each dataset are designated as unknown classes. An unknown class refers to an attack category that is completely withheld from the training and validation data and retained only in the test set for open-set evaluation. All remaining categories, including normal traffic, are treated as known classes. In the main experiments, the selected pair serves as the main reference setting, while several other class combinations are evaluated in Section 4.8 to examine the influence of unknown-class selection. During training, all samples belonging to the designated unknown classes are removed from the training set, and only the remaining known class samples are used for model training and threshold calibration. During testing, the test set contains both known-class samples and samples from the removed unknown attack classes. For known-class samples, the model is required to output the correct known-class labels, whereas unknown samples should be rejected and labeled as unknown. In the implementation, test samples belonging to the designated unknown attack classes are uniformly relabeled as −1 for the calculation of open-set evaluation metrics such as unknown recall and the AUROC.

The specific known- and unknown-class assignments used in the main experiments are summarized in Table 2. In Table 2, the imbalance ratio is calculated by dividing the number of samples in the largest class by that of the smallest class. According to the automatic activation rule, Dual-Reject is enabled when the imbalance ratio is greater than 25; otherwise, only MRCE is used for rejection. Since the known-class distribution of UNSW-NB15 is highly imbalanced, its imbalance ratio reaches 39.9, exceeding the activation threshold of 25. Therefore, the Dual-Reject module is enabled for UNSW-NB15. The imbalance ratio of ToN-IoT is 2.5, which does not exceed the activation threshold, and only MRCE is used. This activation decision relies only on the sample statistics of known classes in the training set and does not use any test-set information.

### 4.2. Experimental Settings and Evaluation Metrics

All experiments use the same model configuration and training procedure. Random forest classifiers are trained separately for the Raw, Graph, 2D-Stat, and Fusion representations. Each classifier contains 200 decision trees, with no maximum depth restriction, a minimum of one sample per leaf node, and balanced_subsample class weights. For the Graph representation, the eight highest-weighted neighbors are retained for each feature node.

To avoid using test-set information for threshold selection, 20% of the known-class training data are selected by stratified sampling as the validation set under each random seed. The mean and standard deviation of the CER scores for each representation are estimated only from the validation set and are used for the Z-score standardization of the test set. The MRCE rejection threshold is set to the 0.8 quantile of the ensemble scores on the validation set. When the imbalance ratio of known classes in the training set is greater than 25, Dual-Reject is further enabled, and the 0.95 quantile of the Mahalanobis scores on the validation set is used as the distance threshold. Although the same quantile levels are used across the experiments, the corresponding numerical rejection thresholds are recalibrated independently for each random seed according to its known-class validation set. During testing, a sample is classified as unknown whenever either the MRCE or Mahalanobis signal exceeds its corresponding threshold.

All experiments are conducted on Ubuntu 20.04.5 using a machine equipped with an Intel(R) Core(TM) i9-10920X CPU @ 3.50 GHz and 62 GiB of RAM. The experimental code is implemented in Python 3.8.10, with the main dependencies including scikit-learn 1.3.2, NumPy 1.23.0, SciPy 1.10.1, pandas 2.0.3, and joblib 1.4.2. For experiments conducted with multiple random seeds, five seeds are used: 7, 42, 168, 123, and 256. The results are reported as the mean ± standard deviation (SD).

The open-set intrusion detection performance of the model is evaluated from three main perspectives: known-class classification performance, unknown-class rejection capabilities, and the ability of the rejection score to distinguish known samples from unknown samples. Accordingly, the Known-F1, unknown recall, the AUROC, and the known-class false rejection rate (FRR) are used as the evaluation metrics. The Known-F1 is used to evaluate the classification performance of the model on known-class samples. Open-set recognition requires the model not only to reject unknown attack samples but also to maintain stable classification performance on known-class samples. The weighted F1-score calculated on known-class test samples is used as the Known-F1, as defined in Equation (11):

(11) $$\mathrm{Known}\text{-}F1=\sum_{c\in Y_{K}}\frac{n_{c}}{|D_{K}|}\cdot \frac{2P_{c}R_{c}}{P_{c}+R_{c}}$$

where $Y_{K}$ is the set of known classes, $D_{K}$ denotes the set of known-class samples in the test set, $n_{c}$ is the number of samples belonging to class *c* among the known test samples, and $P_{c}$ and $R_{c}$, respectively, denote the precision and recall of class *c*. This metric reflects the effect of introducing the rejection mechanism on the known-class classification performance. To further quantify the effect of the rejection mechanism on known samples, the known-class false rejection rate (FRR) is also reported. The FRR is defined as the proportion of known-class test samples that are incorrectly rejected as unknown.

Unknown recall is used to evaluate the rejection capabilities of the model for unknown attack samples, namely the proportion of actual unknown samples that are correctly identified as unknown. In practical intrusion detection scenarios, the missed detection of unknown attacks may result in considerable security risks. Therefore, higher unknown recall indicates a stronger ability to detect attack types that were unseen during training. The AUROC is used to evaluate the overall ability of the rejection score to distinguish known samples from unknown samples. When calculating the AUROC, unknown samples are treated as the positive class and known samples as the negative class, with the output rejection scores used for ranking.

### 4.3. Overall Open-Set Detection Performance

To evaluate the overall open-set detection performance of MRCER-DR under different data distributions, the Known-F1, unknown recall, AUROC, and FRR are reported. According to the imbalance ratio of known classes in the training set, Dual-Reject is automatically enabled for UNSW-NB15, whereas only MRCE is used for ToN-IoT, as shown in Table 3.

MRCER-DR achieves 83.1 ± 0.5% unknown recall, an 86.2 ± 0.1% AUROC, a 76.6 ± 0.1% Known-F1, and a 24.1 ± 0.7% FRR on UNSW-NB15. On ToN-IoT, it achieves 97.7 ± 2.1% unknown recall, a 90.9 ± 0.8% AUROC, an 89.8 ± 0.1% Known-F1, and a 17.8 ± 1.1% FRR. These results provide an overall evaluation of both unknown attack detection and known-class performance under the main open-set settings of the two datasets. To further quantify the uncertainty of the reported mean performance across different random splits, 95% confidence intervals were calculated from the five repeated runs using the t-distribution. On UNSW-NB15, the 95% confidence interval for unknown recall was [82.48%, 83.72%], and the interval for the AUROC was [86.08%, 86.32%]. On ToN-IoT, the 95% confidence interval for unknown recall was [95.09%, 100.00%], while the interval for the AUROC was [89.91%, 91.89%]. The confidence interval bounds were constrained to the valid range of each metric. The relatively narrow confidence intervals for most metrics indicate limited variation across the five repeated runs.

### 4.4. Comparison with Open-Set Baselines

To evaluate the effectiveness of MRCER-DR for open-set intrusion detection, seven representative open-set detection baselines are selected for comparison: MSP-RF, OpenMax, Mahalanobis, Energy-RF, MSP-MLP, Energy-MLP, and varMax [40]. These methods cover commonly used rejection strategies based on the maximum prediction probability, extreme value-based recalibration, feature-space distances, and energy scores. Specifically, MSP-RF uses the maximum class probability from the random forest classifier as its rejection criterion, while MSP-MLP uses the maximum class probability from the multilayer perceptron classifier as its rejection criterion. OpenMax recalibrates class responses according to their deviations from known-class centers. Mahalanobis constructs anomaly scores based on the distances between test samples and known-class feature distributions. Energy-RF and Energy-MLP construct energy-based rejection scores from the outputs of different classifiers. In addition, varMax is a recent zero-day attack recognition method that combines the difference between the two largest class scores, output variance, and an energy score. To ensure a consistent comparison, all methods use the same data splits, feature preprocessing procedures, and open-set class settings. OpenMax, Energy-RF and varMax are adapted to random forest probability outputs according to their respective scoring mechanisms. Mahalanobis distance scores are calculated in the Fusion feature space. The corresponding model statistics and score parameters are estimated using the known-class training or validation data, depending on the requirements of each method. Rejection thresholds are determined from the known-class validation set under a consistent calibration protocol, and the same implementation settings are retained across datasets. No test-set information is used during parameter setting or threshold calibration. The comparison results are shown in Table 4.

As shown in Table 4, the performance of the different baselines varies across the datasets, indicating that the open-set detection performance is jointly affected by the data distribution, the underlying classifier, and the rejection scoring method. For example, Energy-RF achieves the highest unknown recall and AUROC among the baseline methods on UNSW-NB15. On ToN-IoT, varMax achieves the highest baseline performance. These results indicate that the relative performance of different rejection methods is not fully consistent across datasets and that different rejection signals have different applicability characteristics.

Under the same experimental conditions, MRCER-DR achieves the highest unknown recall and AUROC on both datasets. On UNSW-NB15, MRCER-DR improves the unknown recall by 6.1 percentage points and the AUROC by 1.1 percentage points compared with the best-performing baseline. On ToN-IoT, it improves the unknown recall by 11.2 percentage points and the AUROC by 3.8 percentage points. These results indicate that MRCER-DR not only increases the proportion of unknown attack samples detected but also improves the overall discrimination of the rejection scores between known and unknown samples. By jointly utilizing discriminative information from multiple sources, MRCER-DR achieves relatively consistent performance improvements across the main open-set splits of both datasets. To further assess the reliability of these performance differences across different random splits, 95% confidence intervals were calculated from the five repeated runs for MRCER-DR and the best-performing comparison method in Table 4 on each dataset. On UNSW-NB15, the 95% confidence intervals for Energy-RF were [74.89%, 79.11%] for unknown recall and [83.61%, 86.59%] for the AUROC. On ToN-IoT, the corresponding intervals for varMax were [82.15%, 90.85%] and [83.75%, 90.45%]. Compared with the intervals reported for MRCER-DR in Section 4.3, the results indicate a consistent advantage in unknown recall across the repeated runs on both datasets, while the AUROC improvement is smaller but generally maintained.

### 4.5. Ablation Study

To analyze the roles of CER, MRCE, and Dual-Reject in MRCER-DR, MSP rejection is used as the baseline, after which CER, MRCE, and Dual-Reject are introduced sequentially. The unknown recall values of these configurations are compared under the same experimental settings, as shown in Table 5. Dual-Reject is enabled only when the imbalance ratio of known classes is greater than 25. Therefore, the complete MRCER-DR configuration on ToN-IoT is equivalent to the CER + MRCE configuration.

**Figure 2:**
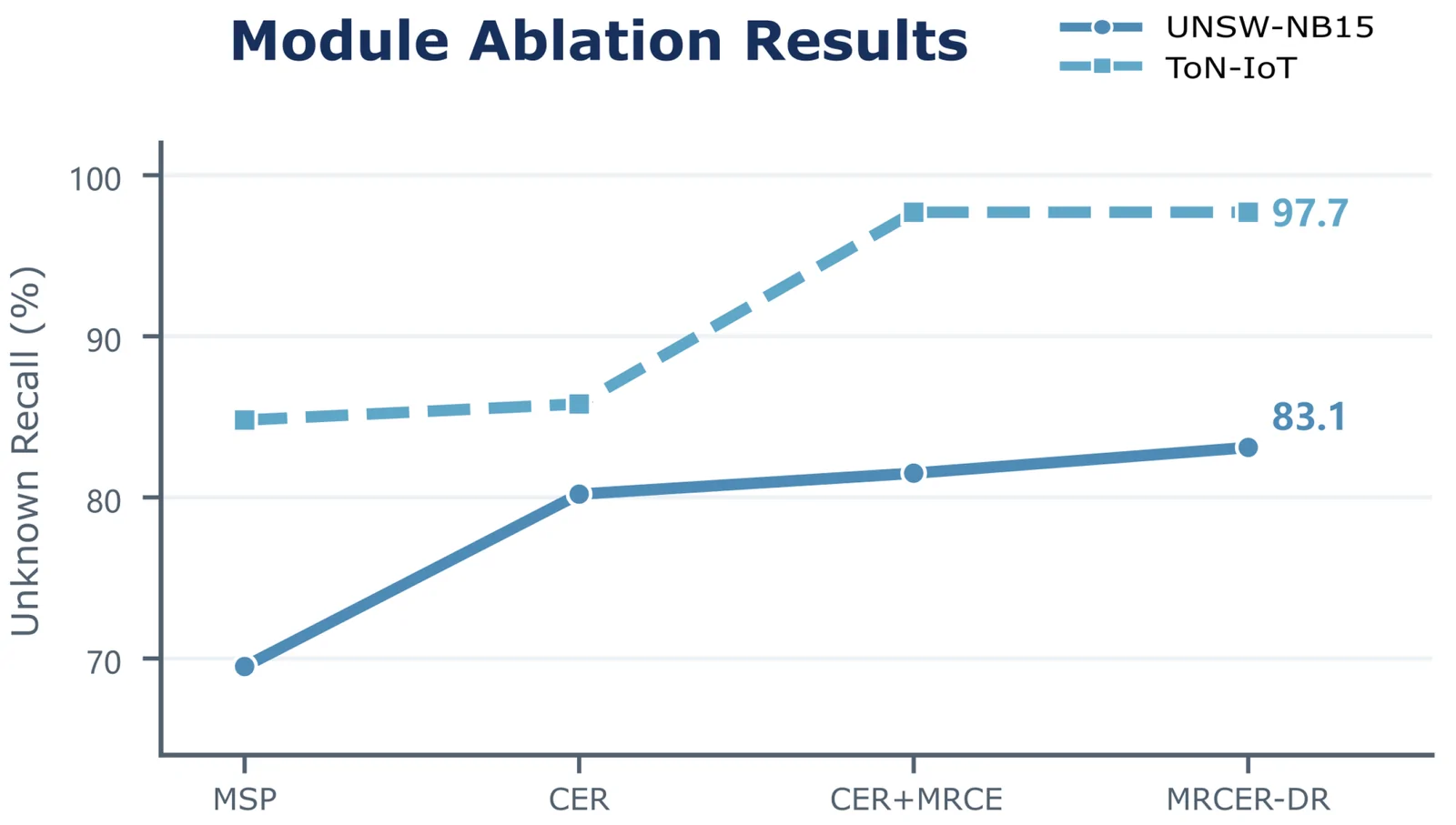
Unknown recall under different module configurations.

On UNSW-NB15, CER increases the unknown recall by 10.7 percentage points, from the MSP result to the CER result. After MRCE is introduced, the unknown recall is further improved, and Dual-Reject increases it to 83.1%. On ToN-IoT, CER improves the unknown recall by 1.0 percentage point over MSP, while MRCE further improves this metric by 11.9 percentage points. The performance trends under different module configurations are shown in Figure 2.

The results show that the contributions of the different modules vary with the data distribution. CER provides the most notable improvement on UNSW-NB15, whereas MRCE is the main source of performance gain on ToN-IoT. Dual-Reject provides an additional improvement only on UNSW-NB15, where the automatic activation condition is satisfied. These results indicate that CER, MRCE, and Dual-Reject contribute under different data conditions. Rather than providing uniform gains, the progressive rejection mechanism improves unknown attack detection by allowing complementary rejection information to compensate for the limitations of a single criterion or representation. CER combines the maximum class probability with the prediction entropy, while MRCE further incorporates uncertainty responses from different representation spaces. Dual-Reject introduces the feature-space deviation as supplementary evidence when probability-based rejection remains insufficient. Therefore, the improvement does not simply result from adding more rejection signals but from combining signals that capture different characteristics of unknown samples.

### 4.6. Analysis of MRCE Representations

To analyze the influence of different representations on the rejection performance, a leave-one-representation-out experiment is conducted. Starting from the complete MRCER-DR configuration, one of the Raw, Graph, 2D-Stat, or Fusion representations is removed from MRCE at a time, and the unknown recall is recalculated while all other experimental settings remain unchanged. The value ΔUR reported in Table 6 represents the performance change relative to the complete configuration after removing the corresponding representation. A negative value indicates a decrease in unknown recall, whereas a positive value indicates an improvement.

As shown in Table 6, the contributions of the different representations vary across the datasets. On UNSW-NB15, removing any representation decreases the unknown recall by 0.4 to 0.9 percentage points, with 2D-Stat having a relatively larger impact. On ToN-IoT, all representations also make positive contributions. Removing Graph causes the largest decrease of 1.9 percentage points. This experiment shows that the roles of different representations are not identical across datasets and that multiple representations can jointly provide useful information for rejection decisions. Therefore, the performance of MRCE largely depends on how information from different representations is combined.

### 4.7. Correlation Analysis of MRCE Scores

To further investigate whether different representations provide complementary rejection information for MRCE, the rank correlations of the CER scores under the Raw, Graph, 2D-Stat, and Fusion representations are analyzed. For any pair of representations, the Spearman correlation coefficients of their CER scores are calculated separately on known and unknown test samples, and the corresponding correlation difference is defined as shown in Equation (12):

(12) $$\Delta \rho =\rho_{\mathrm{known}}-\rho_{\mathrm{unknown}}$$

where $\rho_{\mathrm{known}}$ and $\rho_{\mathrm{unknown}}$ denote the CER score correlation coefficients between the two representations on known and unknown samples. A larger positive Δρ indicates that the two representations have more consistent uncertainty rankings for known samples but show greater ranking differences for unknown samples, suggesting that different representations may capture uncertainty in unknown attacks from different feature perspectives. All correlation coefficients are calculated under five random seeds and then averaged.

**Figure 3:**
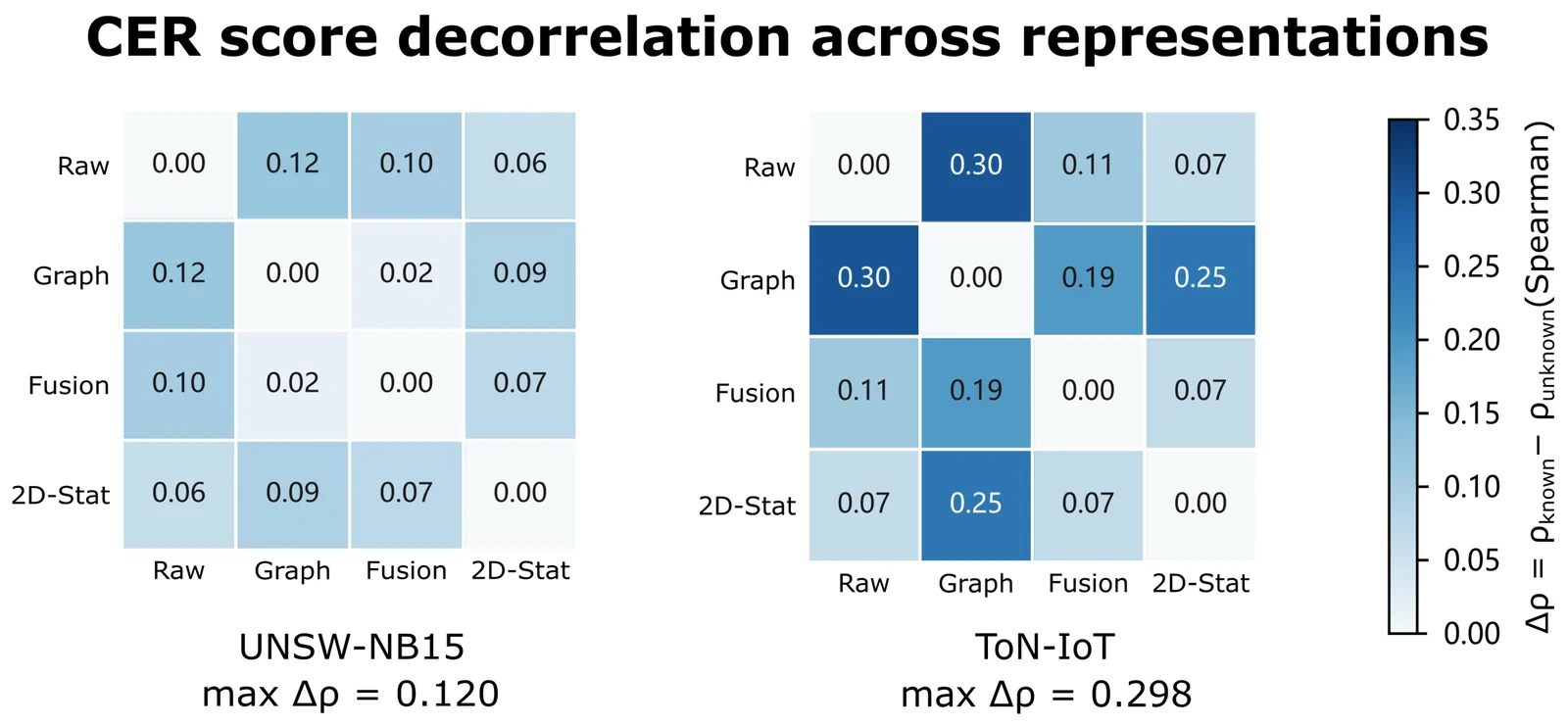
Differences in CER score correlations across representations.

As shown in Figure 3, all representation pairs on both datasets exhibit positive correlation differences, indicating that the CER rankings across different representations are generally less consistent for unknown samples than for known samples. On UNSW-NB15, the overall correlation differences among representations are relatively small. The values for Raw–Graph and Raw–Fusion are 0.120 and 0.101, while those of the remaining pairs range from 0.017 to 0.094. On ToN-IoT, the differences among representations are more pronounced, with values of 0.298, 0.248, and 0.193 for Raw–Graph, Graph–2D-Stat, and Graph–Fusion. These results indicate that the uncertainty rankings of known attack samples are relatively stable across different representation spaces, whereas unknown attacks are more likely to exhibit different response patterns across representations. An unknown sample may show only weak uncertainty in one representation but may exhibit a stronger uncertainty response in another. By integrating the normalized CER scores, MRCE allows such representation-specific rejection information to contribute to the final decision, reducing the dependence on any single feature perspective. This result supports the use of complementary information by MRCE when integrating multi-representation rejection scores. Meanwhile, the correlation differences show a clear dataset dependence. The larger differences on ToN-IoT are mainly concentrated in representation pairs involving Graph, which is generally consistent with the result in Table 6 showing that removing Graph causes the largest decrease in unknown recall. However, a larger Δρ only indicates more pronounced ranking differences between representations for unknown samples and does not necessarily imply that the corresponding representations will improve the final rejection performance. Representation diversity should therefore be considered together with the data distribution, rejection discrimination capabilities, and ensemble strategy.

### 4.8. Unknown-Class Rotation Experiments

To evaluate the sensitivity of MRCER-DR to the selection of unknown classes, three different unknown-class combinations are evaluated on each dataset. For each split, the attack classes listed as unknown in Table 7 are completely withheld from the training and validation data, while all remaining categories are treated as known classes. The model is retrained using only the remaining known classes, and score standardization and threshold calibration are repeated using the known-class validation set. The results are shown in Table 7.

As shown in Table 7, different combinations of unknown classes have some influence on model performance. Across the three UNSW-NB15 settings, the unknown recall ranges from 76.4% to 83.1% and the AUROC ranges from 84.1% to 86.6%, indicating relatively stable overall performance. For ToN-IoT, only MRCE is used under the main open-set split, whereas Dual-Reject is automatically enabled for the other two splits because the known-class imbalance ratio reaches 47.9. Across the three settings, the unknown recall remains between 97.7% and 99.4%, while the AUROC ranges from 90.9% to 98.9%, demonstrating good stability across different unknown-class selections. The experimental results show that the degree of distribution overlap between unknown attacks and known classes affects the rejection difficulty. MRCER-DR maintains good open-set detection performance under most unknown-class rotation settings, although some class dependence remains. However, these within-dataset rotation experiments cannot substitute evaluations of cross-dataset and temporal generalization, and these broader evaluations remain important directions for future work.

### 4.9. Analysis of the Effect of Dual-Reject on Challenging Unknown-Class Detection

**Figure 4:**
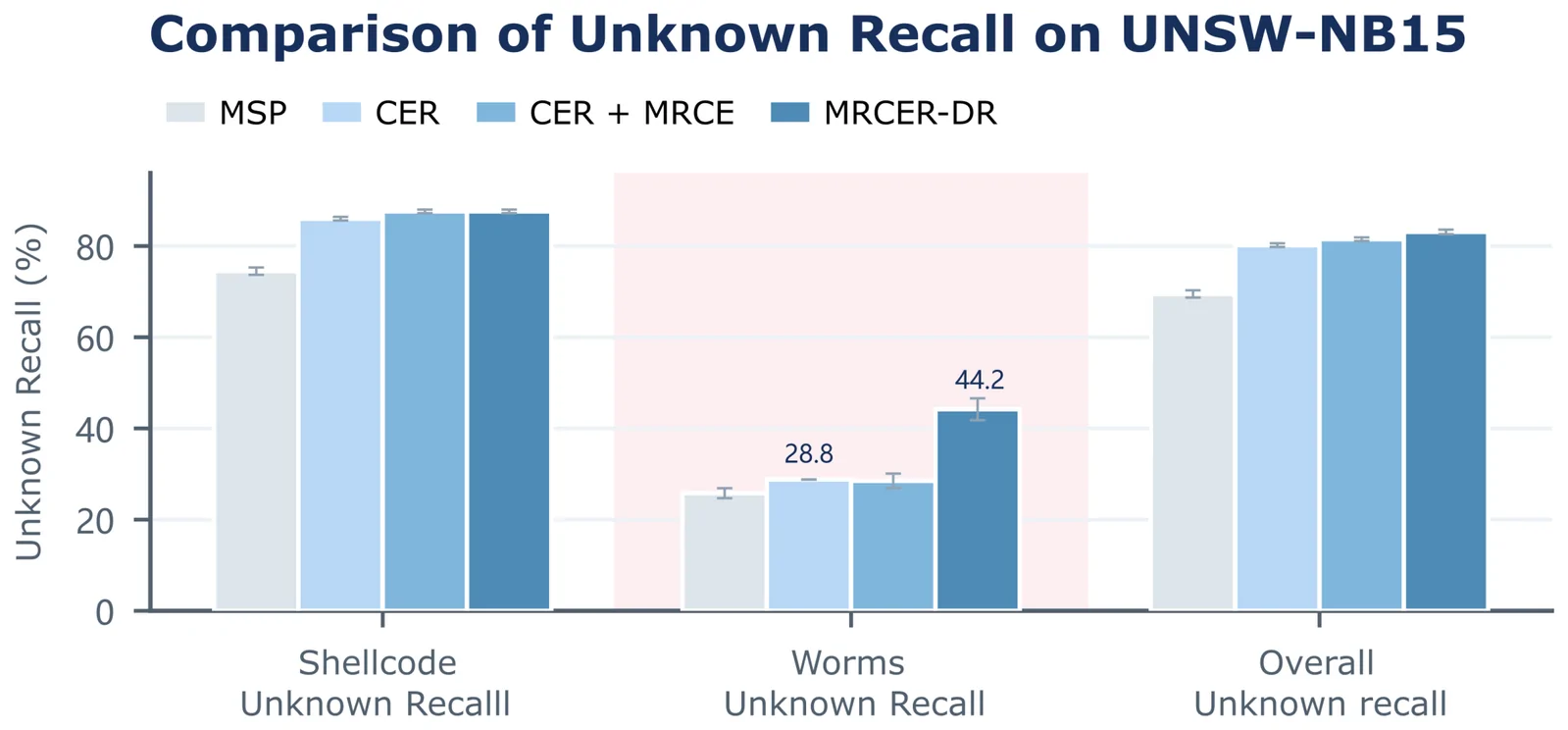
Unknown recall under different rejection configurations on UNSW-NB15.

To analyze the effect of Dual-Reject on challenging unknown classes, the recall of individual unknown classes is compared among MSP, CER, CER + MRCE, and the complete MRCER-DR on UNSW-NB15, where Dual-Reject is enabled. This experiment reports the recall values for shellcode, worms, and all unknown samples to examine the contribution of the Mahalanobis distance signal to the rejection of challenging unknown classes. The experimental results are shown in Figure 4.

As shown in Figure 4, CER and MRCE substantially improve the detection of shellcode, increasing its recall from 74.5% with MSP to 86.0% and 87.6%. However, the improvement for worms is relatively limited, with recall values of 28.8% and 28.5%, compared with 25.8% for MSP. This suggests that worms may strongly overlap with some known classes in the classifier probability space, making it difficult to reject them effectively using only the confidence, prediction entropy, and their multi-representation ensemble. After Dual-Reject is enabled on top of MRCE, the recall for worms increases from 28.5% to 44.2%, an improvement of 15.7 percentage points, while the recall for shellcode remains at 87.6%. Accordingly, the overall unknown recall increases from 81.5% to 83.1%. At the same time, the FRR increases from 20.1% to 24.1%, indicating that the additional Mahalanobis rejection signal improves the detection of challenging unknown samples at the cost of more false rejections of known samples. Since the number of worms test samples is considerably smaller than that of shellcode, the overall unknown recall is calculated over all unknown samples. Therefore, the substantial improvement for worms results in an increase of only 1.6 percentage points in the overall metric. This result indicates that the main benefit of Dual-Reject is not a uniform improvement across all unknown classes but rather the use of an additional feature-space distance signal to identify challenging unknown samples that are difficult for MRCE to cover.

**Figure 5:**
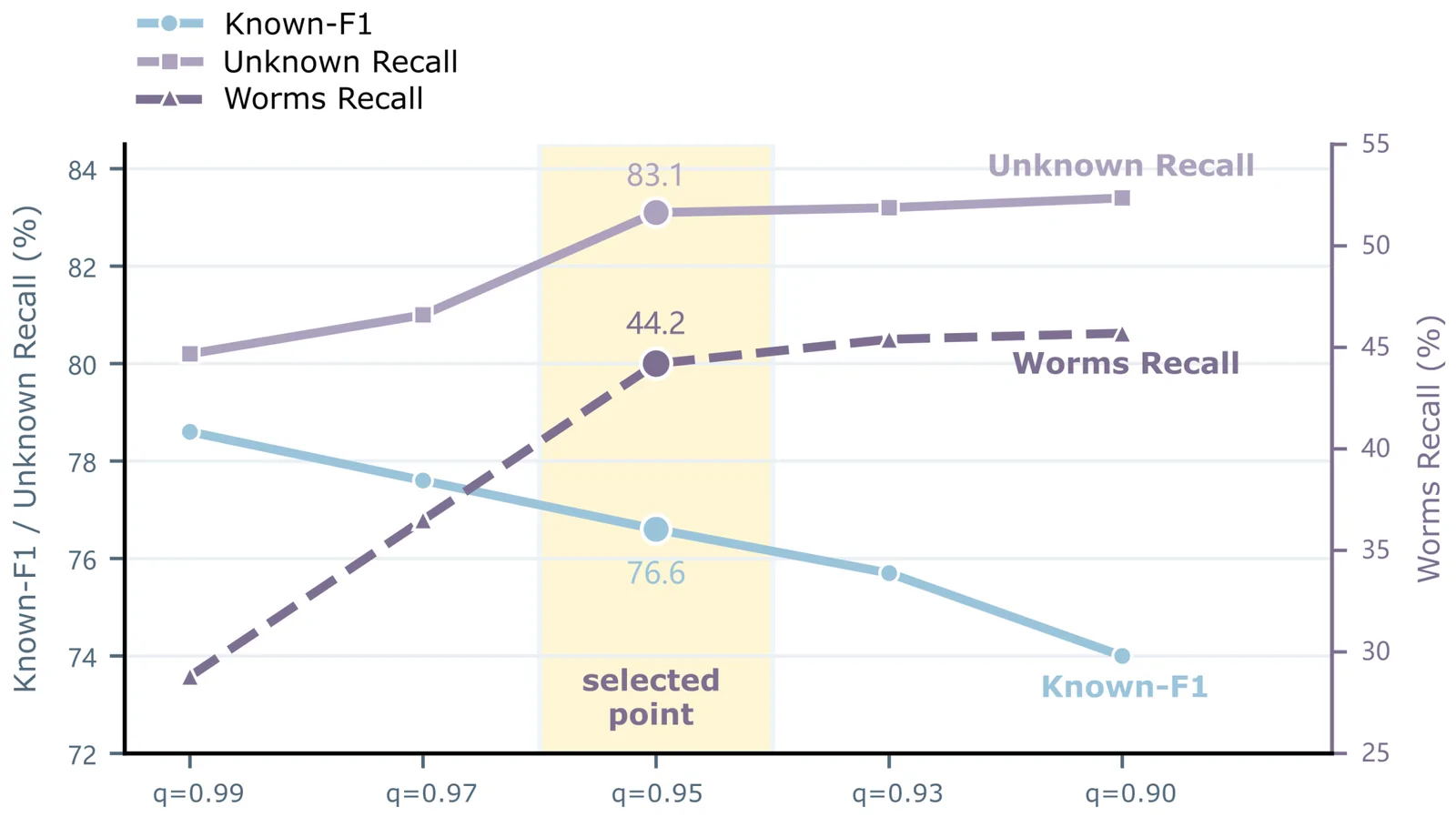
Sensitivity to the Mahalanobis threshold.

The Mahalanobis distance threshold further affects the balance between unknown attack detection and known-class classification performance. To analyze the sensitivity of this parameter, the Mahalanobis threshold quantile on the validation set is set to 0.99, 0.97, 0.95, 0.93, and 0.90 while keeping MRCE, the data splits, and all other experimental settings unchanged. The Known-F1, overall unknown recall, and worms recall under different settings are compared, as shown in Figure 5.

As shown in Figure 5, as the Mahalanobis threshold decreases, the rejection criterion becomes less restrictive. Both the worms recall and overall unknown recall generally increase, while the Known-F1 continuously decreases. This indicates that a lower distance threshold can identify more unknown samples that are missed by probability-based rejection, but it also increases the risk of incorrectly rejecting known samples. When *q* decreases from 0.97 to 0.95, both the worms recall and overall unknown recall show noticeable improvements. Further decreasing *q* to 0.93 and 0.90 produces progressively smaller gains in unknown recall, while the Known-F1 continues to decline. At q=0.95, the model achieves a Known-F1 of 76.6%, an overall unknown recall rate of 83.1%, and worms recall of 44.2%. The adopted value q=0.95 provides a reasonable balance between known-class classification performance and the detection of challenging unknown classes. In addition, this quantile is intentionally stricter than the MRCE setting because the Mahalanobis distance serves only as a supplementary rejection signal under the OR rule. This stricter setting limits additional false rejections of known samples while allowing the distance signal to complement MRCE when challenging unknown samples are not effectively rejected by probability-based information alone.

To further examine whether Dual-Reject should be applied to all datasets, we additionally forced the module to be activated on ToN-IoT, where the imbalance ratio was only 2.5. After Dual-Reject was introduced, the Known-F1 decreased from 89.8 ± 0.1% to 88.4 ± 0.7%, while the unknown recall remained unchanged at 97.7 ± 2.1% and the AUROC remained at 90.9 ± 0.8%. Meanwhile, the FRR increased from 17.8% to 20.1%. This result indicates that introducing the additional Mahalanobis signal under a relatively balanced class distribution does not improve unknown attack detection but increases the false rejection of known samples. In this case, MRCE already identifies most unknown samples, leaving limited room for the Mahalanobis distance to provide further improvement. Therefore, Dual-Reject is conditionally activated rather than applied to all datasets.

Overall, these results show that Dual-Reject can use the Mahalanobis distance to complement the probability-based rejection information provided by MRCE, with its main contribution concentrated on challenging unknown classes such as worms. However, the forced activation experiment on ToN-IoT shows that, when MRCE already identifies most unknown samples, introducing the additional distance criterion provides no improvement in unknown recall while increasing the FRR. These results support the conditional use of Dual-Reject rather than applying it to all datasets. The Mahalanobis threshold sensitivity analysis further shows that the distance threshold affects the balance between challenging unknown-class detection and known-class classification performance. The FRR of 24.1% on UNSW-NB15 indicates that improved unknown attack detection is accompanied by the increased false rejection of known traffic. The practical interpretation of this trade-off depends on how rejection decisions are incorporated into the operational workflow. When rejection is used to flag traffic for further analysis, it mainly serves as an additional screening signal; when it is used to support automatic traffic blocking, the balance between unknown attack detection and false rejection should be considered in relation to the operational requirements of the target scenario.

### 4.10. Rejection Score Threshold Sensitivity Analysis

The selection of the MRCE rejection threshold affects the balance between known-class classification performance and unknown attack detection capabilities. To evaluate the suitability of the threshold setting, we analyze the influence of different threshold quantiles on the open-set detection performance. In the sensitivity analysis, the quantile *q* corresponding to the MRCE rejection threshold is varied from 0.70 to 0.90 on the validation set for both UNSW-NB15 and ToN-IoT. The results are shown in Table 8.

As shown in Table 8, the Known-F1 and unknown recall exhibit the opposite trends as the MRCE rejection threshold quantile increases. On both datasets, a lower threshold quantile increases the recall of unknown attack samples but also causes more known-class samples to be incorrectly rejected, resulting in a lower Known-F1. For example, on UNSW-NB15, when *q* increases from 0.70 to 0.90, the Known-F1 increases from 75.6% to 81.0%, while the unknown recall decreases from 89.3% to 66.7%. These results indicate that a lower rejection threshold improves unknown attack detection but reduces the classification performance on known classes. In contrast, a higher threshold reduces the false rejection of known samples and improves the known-class classification performance but may weaken unknown attack detection. Therefore, the MRCE threshold should balance known-class classification performance and unknown attack detection capabilities. The default setting used in the main experiments lies within the intermediate range of the evaluated quantiles and provides a relatively balanced trade-off between the Known-F1 and unknown recall on both datasets. The sensitivity results therefore support the reasonableness of adopting the 0.8 quantile of the known-class validation MRCE scores as the rejection threshold. The same quantile setting was retained across the five random seeds used in the main experiments, while the numerical threshold was recalibrated from the known-class validation scores for each split. As shown in Table 3, the relatively small standard deviations in Known-F1 and unknown recall indicate stable performance across different splits, while Table 7 further shows that the same setting remains applicable under different unknown-class combinations.

### 4.11. Efficiency Analysis

IoT edge devices are typically constrained by the computational resources and response time. Therefore, in addition to achieving effective detection performance, open-set intrusion detection methods should also limit the training and inference overhead. To evaluate the computational efficiency of MRCER-DR, we measured the training time, test-set prediction time, and single-sample inference latency. The results are shown in Table 9.

As shown in Table 9, the training time of MRCER-DR ranges from 46.12 to 112.73 s across the two datasets, while the total prediction time ranges from 1.75 to 77.88 s. Since the total prediction time is affected by factors such as the number of test samples, the single-sample inference latency provides a more appropriate measure of the practical inference overhead across different datasets. The single-sample inference latency is 1.008 ± 0.313 ms on UNSW-NB15 and 0.043 ± 0.002 ms on ToN-IoT, both remaining below approximately 1.01 ms. The inference overhead on UNSW-NB15 is higher because Dual-Reject is enabled and requires the additional computation of the Mahalanobis distance signal. In contrast, ToN-IoT uses only MRCE, resulting in single-sample latency below 0.05 ms. Although MRCER-DR performs classification and CER score calculation separately for four representations, the representation transformations mainly consist of lightweight statistical operations. CER, MRCE, and Dual-Reject are also primarily implemented as postprocessing procedures based on classifier outputs, without introducing complex deep network structures. Under the current experimental platform and configuration, the method maintains single-sample inference latency at the millisecond level, indicating that it improves the open-set detection capabilities without introducing excessive additional inference overhead and provides a basis for future deployment and evaluation on IoT edge devices.

From a practical deployment perspective, MRCER-DR can be packaged as a single containerized inference service that integrates the required preprocessing components, trained random forest classifiers, and rejection parameters. The four representations, together with the subsequent CER, MRCE, and conditional Dual-Reject procedures, can be executed within the same inference process, avoiding additional communication overhead between separate containers. The trained models and preprocessing parameters can be loaded once when the service starts and reused for subsequent traffic samples, thereby reducing the need for repeated initialization and the model loading overhead. MRCER-DR does not require GPU support and can run entirely on CPU-based environments, avoiding additional GPU-related dependencies in containerized deployment.

In continuous traffic streams, MRCER-DR can process individual flow records. The Raw and 2D-Stat representations are generated through fixed per-record transformations, while the feature relationship graph is determined from the training data and kept fixed during inference for subsequent flow records. The trained models and rejection parameters can be loaded once and reused during continuous processing.

To further evaluate the practical deployment performance of MRCER-DR, we deployed the model on a Raspberry Pi 5 equipped with an Arm Cortex-A76 processor and 8 GB of RAM, using the ToN-IoT dataset generated in an IoT environment for on-device evaluation. The experimental results show that the deployed model occupies 311.4 MB of storage and 594 MB of peak resident memory. Using a single CPU core, the average inference latency is 0.198 ms per flow, corresponding to a throughput of 5058 flows/s. These results indicate that MRCER-DR can maintain low inference latency and high processing throughput on single-board computers such as the Raspberry Pi, supporting its computational feasibility on this class of edge platforms.

## 5. Conclusions and Future Work

This paper proposes MRCER-DR, an open-set intrusion detection method with low inference overhead for IoT environments, to address the difficulties of traditional closed-set intrusion detection methods in identifying attacks that are unseen during training and the limited information provided by single rejection signals and single traffic representations. The proposed method first constructs a basic rejection score using CER by combining the maximum class probability with the prediction entropy. MRCE then integrates uncertainty information from the Raw, Graph, 2D-Stat, and Fusion representations. Finally, according to the degree of imbalance in the known-class training data, the Mahalanobis distance is conditionally introduced to form Dual-Reject, a dual-signal rejection mechanism. The experimental results on the public UNSW-NB15 and ToN-IoT datasets show that MRCER-DR effectively improves unknown attack detection while maintaining relatively stable classification performance on known classes under different data distributions. The leave-one-representation-out and correlation analyses further indicate that different representations provide diverse and complementary rejection information for unknown attack identification. This work extends the use of multi-representation information from conventional feature concatenation or classification result fusion to the level of rejection scores, providing an approach to integrating uncertainty information from multiple sources in open-set intrusion detection. From an application perspective, the proposed method mainly relies on outputs from conventional classifiers, lightweight statistical representations, and postprocessing rejection scores. The experimental results further show low inference overhead and provide evidence of the computational feasibility of deployment in edge computing environments.

However, the experiments are mainly based on two offline public datasets and predefined unknown class splits, which cannot fully reflect complex real network conditions involving continuously evolving attacks, changing traffic distributions, and the gradual emergence of unknown classes. Although the unknown-class rotation experiments in Section 4.8 show relatively stable performance across different unknown attack combinations, cross-dataset and temporal generalization remain to be further investigated. The FRR of 24.1% observed on UNSW-NB15 also represents a practical limitation of the current rejection setting, indicating that the trade-off between unknown attack detection and the false rejection of known traffic should be considered according to the intended operational use. In addition, the current quantile settings for the rejection thresholds and the Dual-Reject activation condition are based on fixed rules. The imbalance ratio threshold of 25 is used as a heuristic activation criterion rather than a universally applicable boundary, while the fixed quantile settings may also require adjustment under different data distributions. The current 2D-Stat representation also uses a fixed feature order for grouped statistical aggregation, and the influence of alternative feature arrangements on the detection performance remains to be further examined.

Future work will focus on three directions: extending the representation set, optimizing edge deployment, and developing adaptive thresholds. First, additional representations that capture different characteristics of network traffic will be introduced beyond the current four representations. For 2D-Stat, alternative feature arrangements and more systematic grouping strategies will also be examined. The complementary relationships among these representations will be further investigated, and the applicability of multi-representation rejection will be assessed on more IoT and industrial Internet datasets, including cross-dataset settings and temporally separated traffic data, to further evaluate the generalization of MRCER-DR under heterogeneous and evolving network environments. Second, for resource-constrained scenarios such as IoT gateways and edge devices, future work will further reduce the computational and storage overhead introduced by multi-representation construction and parallel classifier processing. Representation selection and sparse detection mechanisms based on deployment constraints will be investigated to reduce unnecessary computation while maintaining the detection performance as much as possible. Further deployment evaluations will also be conducted on a broader range of IoT and edge devices with different computational and memory constraints to further assess the practical applicability of MRCER-DR. Finally, future work will investigate adaptive rejection thresholds and Dual-Reject activation strategies for continuously evolving and unlabeled network traffic. The MRCE and Mahalanobis distance thresholds will be dynamically adjusted according to the data distributions and model outputs, while the activation of the supplementary distance branch will be determined adaptively according to the characteristics of the known-class training distribution.

## Figures and Tables

**Table 1:** Comparison of open-set intrusion detection approaches and gaps.

Method Category	References	Main Strategy	Remaining Gap
Output-based rejection	[35,36,37,38,39]	Uses classifier output information to identify unknown samples.	Primarily focuses on uncertainty information within classifier outputs rather than across different representations.
Feature-space rejection	[32,41,42,43]	Identifies unknown samples by measuring their deviation from known-class distributions.	Feature-space rejection alone may provide limited rejection information in complex network traffic environments.
Multi-representation/ multi-view methods	[18,19,20,44,45,46]	Combine multiple representations through evidence aggregation, feature fusion, reconstruction aggregation, or voting.	Fusion of rejection scores and uncertainty responses across representations remains limited.
MRCER-DR	This work	Combines confidence and entropy, integrates CER scores across multiple representations, and conditionally introduces feature-space distance under severe class imbalance.	—

**Table 2:** Open-set class assignments and rejection mode settings.

Dataset	Known Classes	Unknown Classes	Imbalance Ratio	Activated Rejection Mode
UNSW-NB15	normal, generic, exploits, fuzzers, dos, reconnaissance, analysis, backdoors	worms, shellcode	39.9	MRCE + Dual-Reject
ToN-IoT	normal, ddos, dos, injection, password, ransomware, scanning, xss	mitm, backdoor	2.5	MRCE

**Table 3:** Overall open-set detection performance of MRCER-DR (mean ± SD over five random seeds).

Dataset	Rejection Mode	Known-F1 (%)	Unknown Recall (%)	FRR (%)	AUROC (%)
UNSW-NB15	MRCE + Dual-Reject	76.6 ± 0.1	83.1 ± 0.5	24.1 ± 0.7	86.2 ± 0.1
ToN-IoT	MRCE	89.8 ± 0.1	97.7 ± 2.1	17.8 ± 1.1	90.9 ± 0.8

**Table 4:** Performance comparison of different open-set detection methods.

Method	UNSW-NB15 (UR/AUROC) (%)	ToN-IoT (UR/AUROC) (%)
MSP-RF	69.5/83.2	84.8/85.5
OpenMax	68.4/83.4	85.8/85.2
Mahalanobis	64.3/82.8	82.3/83.7
Energy-RF	77.0/85.1	84.8/85.4
MSP-MLP	65.9/83.3	80.4/81.6
Energy-MLP	69.0/84.9	81.2/82.1
varMax	73.9/83.9	86.5/87.1
MRCER-DR	83.1/86.2	97.7/90.9

**Table 5:** Module ablation results of MRCER-DR (mean ± SD over five random seeds).

Dataset	MSP (%)	CER (%)	CER + MRCE (%)	MRCER-DR (%)
UNSW-NB15	69.5 ± 0.8	80.2 ± 0.4	81.5 ± 0.4	83.1 ± 0.5
ToN-IoT	84.8 ± 2.8	85.8 ± 1.3	97.7 ± 2.1	97.7 ± 2.1

**Table 6:** Leave-one-representation-out results for MRCE (full MRCER-DR UR: mean ± SD over five random seeds).

Dataset	Full MRCER-DR UR (%)	Without Raw ΔUR (pp)	Without Graph ΔUR (pp)	Without 2D-Stat ΔUR (pp)	Without Fusion ΔUR (pp)
UNSW-NB15	83.1 ± 0.5	−0.4	−0.5	−0.9	−0.2
ToN-IoT	97.7 ± 2.1	−0.8	−1.9	−0.1	−0.7

**Table 7:** Detection performance under different unknown-class settings (mean ± SD over five random seeds).

Dataset	Unknown Classes	IR	Mode	Known-F1 (%)	Unknown Recall (%)	AUROC (%)
UNSW-NB15	worms, shellcode	39.9	MRCE + Dual-Reject	76.6 ± 0.1	83.1 ± 0.5	86.2 ± 0.1
	backdoors, analysis	533.6	MRCE + Dual-Reject	76.8 ± 0.9	80.9 ± 0.6	84.1 ± 1.6
	reconnaissance, dos	533.6	MRCE + Dual-Reject	80.5 ± 1.1	76.4 ± 0.3	86.6 ± 0.8
ToN-IoT	mitm, backdoor	2.5	MRCE	89.8 ± 0.1	97.7 ± 2.1	90.9 ± 0.8
	ransomware, xss	47.9	MRCE + Dual-Reject	89.7 ± 0.3	99.0 ± 0.5	97.4 ± 0.9
	injection, scanning	47.9	MRCE + Dual-Reject	91.8 ± 1.9	99.4 ± 0.4	98.9 ± 0.7

**Table 8:** Open-set detection performance under different MRCE rejection threshold quantiles.

Dataset	Quantile *q*	Known-F1 (%)	Unknown Recall (%)
UNSW-NB15	0.70	75.6	89.3
	0.75	77.6	85.7
	0.80	79.1	81.5
	0.85	80.3	75.6
	0.90	81.0	66.7
ToN-IoT	0.70	88.0	98.9
	0.75	88.9	98.1
	0.80	89.8	97.7
	0.85	90.4	95.4
	0.90	92.2	86.4

**Table 9:** Training and inference efficiency of MRCER-DR (mean ± SD over five random seeds).

Dataset	Training Time (s)	Prediction Time (s)	Inference Latency (ms/Sample)
UNSW-NB15	112.73 ± 5.26	77.88 ± 24.15	1.008 ± 0.313
ToN-IoT	46.12 ± 2.32	1.75 ± 0.09	0.043 ± 0.002

## Data Availability

The data presented in this study are openly available in the following repositories: UNSW-NB15 at https://research.unsw.edu.au/projects/unsw-nb15-dataset (accessed on 6 October 2026), TON-IoT at https://ieee-dataport.org/documents/toniot-datasets (accessed on 6 October 2026).

## Appendix A. Evaluation Using the Official UNSW-NB15 Train/Test Partition

To further evaluate the influence of the data splitting protocol, we conducted experiments using the official data split of the UNSW-NB15 dataset. The same preprocessing procedure, model configuration, open-set setting, and five random seeds were used as in the main experiments. The results are shown in Table A1.

**Table A1:** Performance of MRCER-DR using the official UNSW-NB15 data split (mean ± SD over five random seeds).

Dataset	Known-F1 (%)	Unknown Recall (%)	AUROC (%)
UNSW-NB15	74.8±0.7	80.9±0.5	85.7±0.3

Compared with the main experimental settings, the official UNSW-NB15 split yields a Known-F1 of 74.8%, 1.8 percentage points lower than 76.6%; unknown recall of 80.9%, 2.2 percentage points lower than 83.1%; and an AUROC of 85.7%, 0.5 percentage points lower than 86.2%.
